# Exploring the Effects of Pulsed Electric Field Processing Parameters on Polyacetylene Extraction from Carrot Slices

**DOI:** 10.3390/molecules20033942

**Published:** 2015-03-02

**Authors:** Ingrid Aguiló-Aguayo, Corina Abreu, Mohammad B. Hossain, Rosa Altisent, Nigel Brunton, Inmaculada Viñas, Dilip K. Rai

**Affiliations:** 1 IRTA, XaRTA-Postharvest, Edifici Fruitcentre, Parc Científic i Tecnològic Agroalimentari de Lleida, Lleida 25003, Catalonia, Spain; 2 Department of Food Biosciences, Teagasc Food Research Centre Ashtown, Dublin 15, Ireland; 3 School of Agriculture and Food Science, University College Dublin, Dublin 4, Ireland; 4 Food Technology Department, University of Lleida, XaRTA-Postharvest, Agrotecnio Center, Rovira Roure 191, Lleida 25198, Catalonia, Spain

**Keywords:** pulsed electric fields, polyacetylenes, falcarinol, falcarindiol, carrot, RSM

## Abstract

The effects of various pulsed electric field (PEF) parameters on the extraction of polyacetylenes from carrot slices were investigated. Optimised conditions with regard to electric field strength (1–4 kV/cm), number of pulses (100–1500), pulse frequency (10–200 Hz) and pulse width (10–30 μs) were identified using response surface methodology (RSM) to maximise the extraction of falcarinol (FaOH), falcarindiol (FaDOH) and falcarindiol-3-acetate (FaDOAc) from carrot slices. Data obtained from RSM and experiments fitted significantly (*p* < 0.0001) the proposed second-order response functions with high regression coefficients (R^2^) ranging from 0.82 to 0.75. Maximal FaOH (188%), FaDOH (164.9%) and FaDOAc (166.8%) levels relative to untreated samples were obtained from carrot slices after applying PEF treatments at 4 kV/cm with 100 number of pulses of 10 μs at 10 Hz. The predicted values from the developed quadratic polynomial equation were in close agreement with the actual experimental values with low average mean deviations (*E*%) ranging from 0.68% to 3.58%.

## 1. Introduction

The presence of falcarinol-type polyacetylenes in carrots has attracted considerable interest in recent times due to their potential beneficial effects for the human health [1]. Falcarinol (FaOH) is the most biologically active compound of three main polyacetylenes present in carrots with pronounced cytotoxic activity against several cancer lines [2]. Falcarindiol (FaDOH) possesses cytotoxic [1,3,4] and anti-mutagenic [5] activity *in vitro*, although it appears to be less bioactive than FaOH. Furthermore, FaDOH is an effective inhibitor of cyclooxygenases (COX), in particular COX-1, whereas the anti-COX activity of FaOH does not seem to be pronounced [6,7]. Pharmacological inhibition of COX can provide relief from the symptoms of inflammation and pain. Falcarindiol-3-acetate (FaDOAc) is less active than either FaOH or FaDOH. In fact, very little has been reported regarding its function, however it is always found present with other falcarinol type polyacetylenes in plants, and may exhibit some synergistic anti-fungal effects in plant [8].

Despite the fact that the falcarinol-type polyacetylenes have been the subject of many scientific studies showing to a wide range of their potential health-beneficial bioactivities, it is however not possible to purchase commercial authenticated standards of these compounds due to their low abundance in natural sources [9]. Therefore it is essential that the methods of extraction, enrichment and isolation of polyacetylenes from the natural sources are aligned to generate highest yields of these compounds. In addition, falcarinol-type polycetylenes are thermally unstable and are susceptible to photodecomposition [10]; it is for this reason that the polyacetylenes are usually extracted in low light and at low temperatures. Furthermore, polyacetylenes are lipophilic and therefore are generally extracted using non-polar organic solvents by conventional solid-liquid extraction. An automated pressurized liquid extraction system which also utilises organic solvents but has the advantages of light avoidance and temperature control has also been reported [11]. Pulsed electric fields (PEF) on the other hand have been primarily studied as a novel non-thermal and cost-effective tool in food safety. However, in recent years there has been increasing application of PEF to extract intracellular metabolites of commercial interest in a high throughput and efficient manner. The underlying mechanism of PEF action is that it induces electroporation of cell membranes, increasing the cell-membrane permeability and release of valuable compounds [12,13]. PEF with very short duration (generally from several microseconds to milliseconds) and moderate electric field strengths of 0.5–7 kV/cm have shown to cause a high level of tissue disintegration [14,15,16]. Our previous study on carrot puree has shown the effectiveness of PEF pre-treatment to enhance polyacetylene extraction [17]. However, there is little information in the literature on the optimization of PEF processing parameters to maximise extraction of polyacetylenes from carrots. Moreover, the anatomical location of polyacetylenes in carrots has demonstrated that the highest concentrations of polyacetylenes were present in the phloem and the upper part of the root [18]. Therefore, the aim of this research was to study the effect of electric field strength, number of pulses, pulse frequency and pulse width on the main polyacetylenes (FaDOH, FaDOAc and FaOH) in carrot slices. Moreover, an optimization of PEF processing parameters was carried out in order to extract the highest amount of these polyacetylenes.

## 2. Results and Discussion

### 2.1. Effect of PEF Processing Parameters on Polyacetylenes

The levels of individual polyacetylenes in unprocessed carrot slices after pressurized liquid extraction were 55.20 μg/g DW for FaOH, 71.37 μg/g DW for FaDOH and 28.29 μg/g DW for FaDOAc. Studies on carrots have shown that FaOH levels extracted could vary from 20 to 359 μg/g depending on carrot cultivar [8,18,19,20]. Values of FaDOH and FaDOAc were in the range reported by Rawson *et al.* [9] in fresh peeled carrot. Effect of PEF processing on the relative contents of falcarinol (FaOH), falcarindiol (FaDOH) and falcarindiol-3-acetate (FaDOAc) are shown in Table 1. The maximum FaOH (210.2%), FaDOH (179%) and FaDOAc (169.6%) contents were observed when applying 100 number of pulses of 10 μs at 10 Hz with an electric field strength of 4 kV/cm compared to untreated carrot slices. Thus, the pre-treatment of carrot slices with PEF processing was effective by almost 100% to enhance polyacetylene extraction in combination with pressurized liquid extraction.

**Table 1:** Central composite response surface design for polyacetylenes on carrot slices treated under different mild PEF treatment conditions.

Mild PEF Treatment Conditions *						Relative Content (%) **^†^		
Electric Field (kV/cm)	Number of Pulses	Pulse Frequency (Hz)	Pulse Width (μs)	Specific Energy (KJ/kg)	Increase of Temperature	FaOH	FaDOH	FaDOAc
1	100	10	10	0.1	0.05 ± 0.01	169.1 ± 8.0	130.4 ± 8.0	128.7 ± 8.2
4	100	10	10	2.4	0.90 ± 0.10	210.2 ± 11.0	179 ± 12.1	169.6 ± 10.4
1	1500	10	10	2.2	1.05 ± 0.20	190.8 ± 4.0	159.0 ± 3.8	115.4 ± 2.0
4	1500	10	10	35.6	6.00 ± 0.50	193.9 ± 2.0	88.6 ± 2.4	119.4 ± 3.0
1	100	200	10	0.1	0.06 ± 0.01	209.8 ± 1.0	135.7 ± 4.7	121.0 ± 1.3
4	100	200	10	2.4	1.00 ± 0.15	130.9 ± 2.0	166.3 ± 3.0	139.8 ± 2.5
1	1500	200	10	2.2	1.50 ± 0.22	140.0 ± 2.1	120.5 ± 7.0	70.5 ± 2.0
4	1500	200	10	35.6	6.40 ± 0.30	115.6 ± 8.0	97.4 ± 5.0	116.8 ± 4.3
1	100	10	30	0.4	0.31 ± 0.05	150.7 ± 2.0	142.7 ± 4.5	119.1 ± 3.2
4	100	10	30	7.1	1.30 ± 0.15	134.1 ± 6.0	126.0 ± 4.2	115.9 ± 4.3
1	1500	10	30	6.7	1.10 ± 0.14	127.8 ± 12.0	117.1 ± 6.5	99.3 ± 8.0
4	1500	10	30	106.7	10.0 ± 0.5	102.1 ± 7.2	73.6 ± 5.1	125.5 ± 4.0
1	100	200	30	0.4	0.05 ± 0.10	147.6 ± 3.2	150.2 ± 2.1	88.7 ± 1.3
4	100	200	30	7.1	1.00 ± 0.10	111.5 ± 5.4	125.5 ± 4.0	94.2 ± 2.0
1	1500	200	30	6.7	1.20 ± 0.10	122.8 ± 6.0	128.2 ± 9.1	115.6 ± 7.2
4	1500	200	30	106.7	10.0 ± 0.5	97.2 ± 5.0	72.8 ± 3.0	67.4 ± 2.5
1	800	105	20	2.4	2.20 ± 0.10	139.3 ± 1.4	139.9 ± 4.4	100.6 ± 4.2
4	800	105	20	37.9	6.20 ± 0.22	100.5 ± 3.0	119.3 ± 3.7	124.3 ± 2.0
2.5	100	105	20	1.9	0.65 ± 0.10	131.1 ± 4.2	130.7 ± 3.2	105.5 ± 3.5
2.5	1500	105	20	27.8	5.20 ± 0.20	57.4 ± 10.0	109.0 ± 9.5	48.2 ± 9.4
2.5	800	10	20	14.8	3.00 ± 0.32	106.0 ± 9.3	102.0 ± 8.1	86.0 ± 6.1
2.5	800	200	20	14.8	3.20 ± 0.50	104.8 ± 5.5	65.5 ± 8.3	59.1 ± 3.0
2.5	800	105	10	7.4	1.60 ± 0.20	82.7 ± 6.2	91.8 ± 6.0	92.0 ± 3.0
2.5	800	105	30	22.2	3.30 ± 0.10	96.1 ± 5.2	66.4 ± 3.0	81.8 ± 4.0
2.5	800	105	20	14.8	2.60 ± 0.30	102.7 *** ± 12.0	110.2 *** ± 11.3	78.5 *** ± 11.0

Notes: * Order of the assays was randomized; ** Data shown are the mean ± SD of 3 treatment repetitions, each assay was performed in triplicate; *** Data shown are the mean of 6 repetitions; ^†^ 100% of retention corresponds to concentrations of FaOH, FaDOH and FaDOAc to 55.20 ± 2.0, 71.37 ± 5.1, 28.29 ± 4.0, µg/g of DW, respectively

Results of the analysis of variance (F-test) for each dependent variable and their corresponding coefficients of determination (R^2^) obtained by fitting the second-order response model to the experimental data are shown in Table 2. The ANOVA indicates that a second order model described with accuracy the changes in the relative content of polyacetylenes of PEF-treated carrot slices (*p* < 0.0001) (Table 2). The determination coefficients (R^2^) were 0.75, 0.81 and 0.82 for FaOH, FaDOH and FaDOAc, respectively, and the lack-of-fit was not significant, meaning that the models were adequate for predicting the response across the design space.

**Table 2:** Analysis of variance of the second-order models for the polyacetylenes in mild PEF-treated carrot slices.

	F-Value		
**Source ^1^**	FaOH	FaDOH	FaDOAc
**Quadratic Model**	11.74 ***	11.65 ***	14.79 ***
**E**	5.11 *	8.33 *	4.20 *
***n***	7.68 **	27.85 ***	13.81 *
***f***	5.24 *	1.25	13.97 *
***τ***	15.64 **	7.48 **	9.06 **
**E^2^**	32.25 ***	10.58 **	50.16 ***
***n*^2^**	0.11	3.86	0.04
***f*^2^**	1.28	1.83	0.43
***τ*^2^**	0.05	7.23 *	0.82
**E · *n***	3.50	16.14 **	0.32
**E · *f***	4.00 *	0.03	0.59
**E · τ**	0.25	4.83 *	6.25 *
***n* · *f***	0.70	1.01	0.05
***n* · τ**	0.03	0.05	5.97 *
***τ* · *f***	2.27	1.34	0.02
**R^2^**	0.75	0.81	0.82
***Adjusted* R^2^**	0.69	0.75	0.77

^1^ E = Electric field strength; *n* = number of pulses; *f* = pulse frequency; τ = pulse width; FaOH = Falcarinol; FaDOH = Falcarindiol; FaDOAc = Falcarindiol-3-acetate; * significant at *p* < 0.05; ** significant at *p* < 0.01; *** significant at *p* < 0.0001.

#### 2.1.1. Falcarinol (FaOH)

The relative FaOH content was represented by polynomial quadratic equations in terms of the studied PEF parameters (Equation (1)):

(1) $$FaOH(\%)=290.3-98.8\cdot E-0.02\cdot n+0.064\cdot f-1.962\cdot \tau -0.073\cdot E\cdot f+19.8\cdot E^{2}$$

where *E* is the electric field strength (kV/cm), *n* the number of pulses, *f* is the pulse frequency (Hz) and τ is the pulse width (μs).

The number of pulses and pulsed width had a significant effect (*p* < 0.01) on the FaOH content of carrot (Table 2). The linear coefficients of these parameters were negative, meaning that the FaOH content depleted with increasing the duration of the treatment.

**Figure 1:**
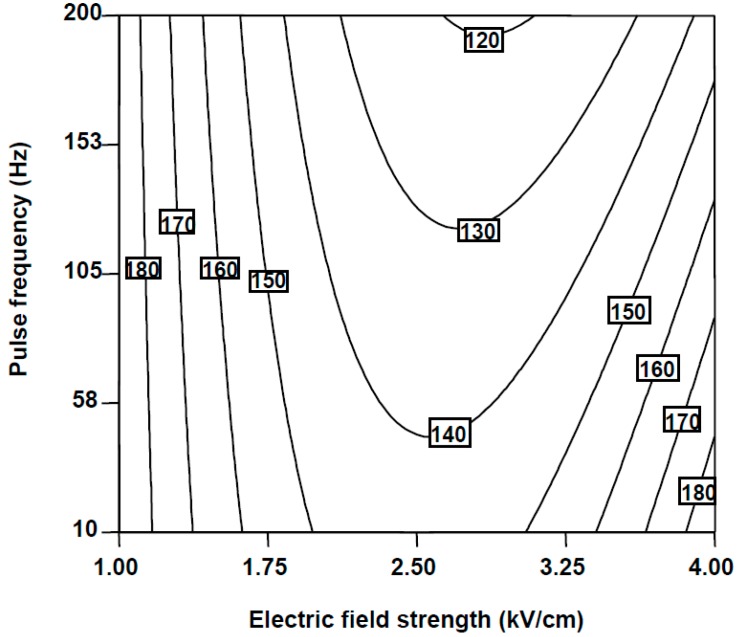
Counter plots for the combined effect of pulse frequency and electric field strength on FaOH relative content of carrot slices treated with 100 number of pulses at pulse widths of 10 μs.

Moreover, the positive value of the quadratic term of *E* (*p* < 0.0001) (Table 2) indicated that relative FaOH content reached a minimum as electric field strength rose. For example, PEF treatments carried out at 10 Hz and electric field strengths from 2.68 to 3.14 KV/cm led to the minimum residual FaOH values of 120% using 100 pulses of 10 μs (Figure 1).

Nevertheless, the electric field strength and pulse frequency also had an important effect on the FaOH levels extracted from carrot slices (*p* < 0.05). As a result of the synergy between these parameters (*p* < 0.05), it was suitable to accurately define optimum values within the range of studied conditions to achieve the highest levels of FaOH content. It was possible to predict maximum relative FaOH values of 188% when applying 4 kV/cm at 10Hz or 1 kV/cm at 94 Hz keeping constant number of pulses at 100 and pulse widths of 10 μs (Figure 1). Previous studies in carrot puree observed that the highest levels of FaOH detected in PEF-treated carrot purees were up to 1.92-fold higher than untreated samples when processing at 0.25 kV/cm and 2000 pulses of 20 μs at 10 Hz [17]. The behaviour of polyacetylenes to PEF processing could be related to the fact that FaOH is a precursor of both the FaDOH and the FaDOAc and, therefore, in the presence of active enzyme systems, the compounds may be interconverted [8,19]. However, high-intense and short PEF conditions have been reported to be effective in extracting other bioactive compounds. For instance, López, Puértolas, Condón, Raso and Alvarez [21] reported that the application of five pulses at 7 kV/cm increased 4-fold the total amount of betainin recovered from red beetroot compared to untreated samples.

#### 2.1.2. Falcarindiol (FaDOH)

The relative FaDOH content was represented by polynomial quadratic equation in terms of the studied PEF parameters (Equation (2)):

(2) $$FaDOH(\%)=113.1-46.5\cdot E-0.049\cdot n+9.520\cdot \tau -0.014\cdot E\cdot n-0.524\cdot E\cdot \tau +12.28\cdot E^{2}-0.224\cdot \tau^{2}$$

where *E* is the electric field strength (kV/cm), *n* the number of pulses, *f* is the pulse, frequency (Hz) and τ is the pulse width (μs).

**Figure 2:**
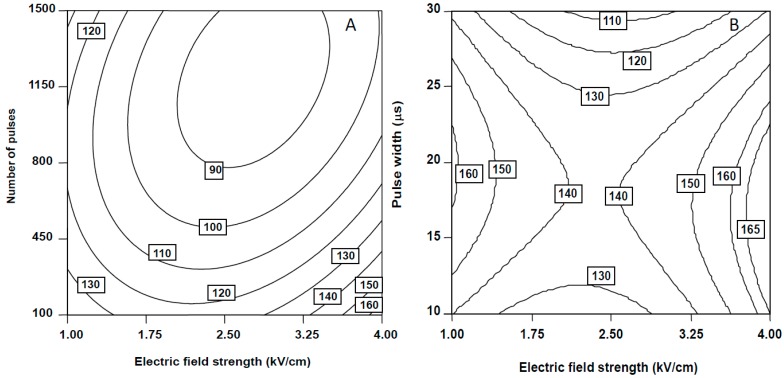
Counter plots for the combined effect of the number of pulses and electric field strength (**A**) and the combined effect of pulse width and electric field strength (**B**) on FaDOH relative content of carrot slices. (A) Pulse frequency set at 10 Hz and pulse width set at 10 μs. (B) Pulse frequency set at 10 Hz and number of pulses set at 100.

Electric field strength (*p* < 0.05), number of pulses (*p* < 0.0001) and pulse width (*p* < 0.01) affected linearly the relative FaDOH content, whereas pulse frequency did not exert influence (*p* > 0.05) on the levels of FaDOH extracted from carrot slices. The positive quadratic terms describing the effects of electric field strength (p < 0.01) and pulse width (*p* < 0.05) on the FaDOH content were also significant. However, the interaction terms *E·n* and *E·τ* indicated that the effect of electric field strength was clearly influenced by the number of pulses and the pulse width (Table 2). The coefficient sign of these interactions were negative; thus indicating that these factors acted in opposite directions. The simultaneous increase of electric field strength from 2.5 to 4.0 kV/cm and the decrease of 10 μs of pulses from 450 to 100, resulted in an increment from 120% to 173% on the relative amounts of FaDOH at frequency of 10 Hz (Figure 2A). As pulse frequency is not a dependent factor and high levels would increase the cost of extraction, pulse frequency was kept at the lowest level of 10 Hz. Moreover, it was possible to exchange different combinations of the variables *E·n* and *E·τ* to extract the same level of FaDOH (Figure 2). Thus, the maximum FaDOH levels were obtained by combining electric field strengths of 4 kV/cm with 100 number of pulses and pulse widths lower than 12 μs (Figure 2B). As a result of these PEF treatment conditions, an increase in the membrane electroporation and an improvement in mass transfer might have induced, which led the increase of polyacetylenes extractability from carrot slices [22]. Aguiló-Aguayo *et al.* [17] also reported an increase of three times the amount of FaDOH extracted from untreated carrot puree when applying 0.25 kV/cm, 100 number of pulses of 20 μs at 10 Hz.

#### 2.1.3. Falcarindiol-3-acetate (FaDOAc)

Relative FaDOAc experimental values were modelled through the polynomial Equation (3), where *E* is the electric field strength (kV/cm), *n* the number of pulses, *f* is the pulse frequency (Hz) and τ is the pulse width (μs).

(3) $$FaDOAc(\%)=198.3-61.1\cdot E-0.04\cdot n-0.12\cdot f-0.474\cdot \tau -0.54\cdot E\cdot \tau +1.13\cdot 10^{-3}\cdot n\cdot \tau +15.21\cdot E^{2}$$

**Figure 3:**
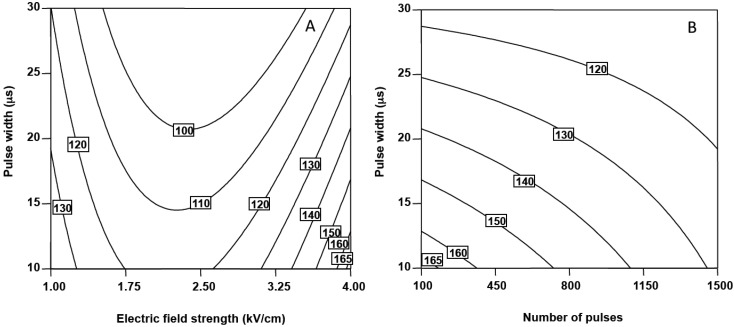
Counter plots for the combined effect of pulse width and electric field strength (**A**) and pulse width and number of pulses (**B**) on FaDOAc relative content of carrot slices. (A) Pulse frequency set at 10 Hz and number of pulses set at 100. (B) Pulse frequency set at 10 Hz and electric field strength set at 4 kV/cm.

As can be seen, the linear terms of the equation for electric field strength, number of pulses, frequency and pulse width (*p* < 0.05) were negative, indicating that increasing these parameters led to the lowest relative FaDOAc contents. Nevertheless, the positive value of the quadratic term of the electric field strength (*p* < 0.0001) indicated that FaDOAc retention reached a minimum as this parameter increase. In fact, increasing the electric field strengths up to 3 kV/cm led to reductions in the amount of FaDOAc extracted from carrot (Figure 3A). Aguiló-Aguayo *et al.* [17] observed that treating carrot puree at 1 kV/cm and 500 pulses of 20 μs reduced the FaDOH and FaDOAc content up to 80% less with respect to untreated carrot purees.

The effect of pulse width was affected by the electric field strength and the number of pulses as indicated by the significance (*p* < 0.05) of the interaction terms E·τ and n·τ (Table 2). The negative sign of the interaction E·τ indicated that combination of high electric field strengths and short pulse widths led to the high FaDOAc retentions (Figure 3A). Figure 3B confirms that maximum permeabilisation could be achieved in carrot slices after PEF treatments consisting of 100 pulses of 10 μs resulting in greatest recovering of FaDOAc (167%) setting pulse frequency at 10 Hz and electric field strength at 4 kV/cm.

### 2.2. Optimization and Model Validation

**Figure 4:**
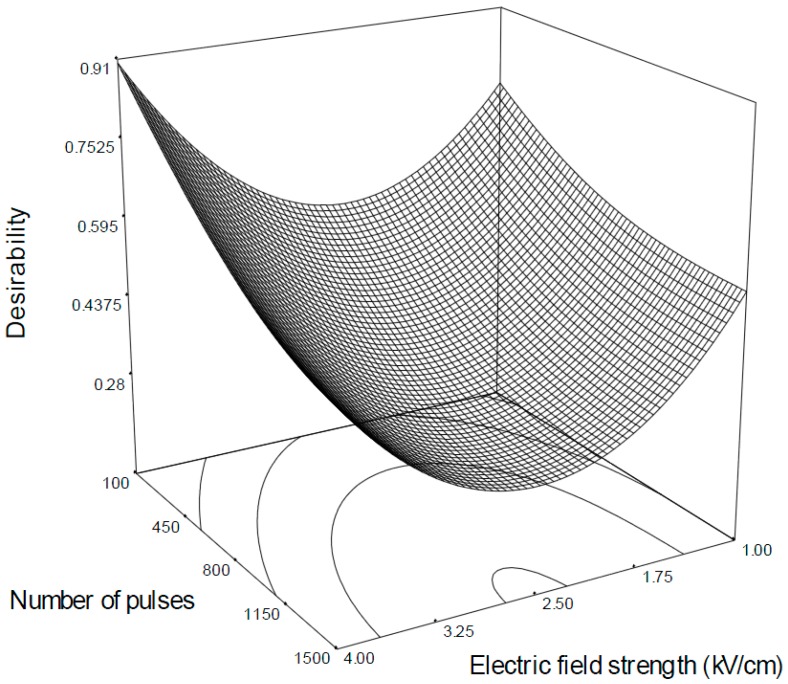
Response surface plot desirability of mild PEF-treated carrot slices as a function of maximal FaOH, FaDOH and FaDOAc retention keeping constant pulse frequency at 10 Hz and pulse width for 10 μs.

Optimal PEF treatment conditions for maximizing the amounts of polyacetylenes extracted from carrot slices were determined. To this purpose, the same priority was assigned to each variable, seeking maximum levels of FaOH, FaDOH and FaDOAc. The desirability was achieved by combining low number of pulses and high electric field strengths (Figure 4). An overall score of 0.910 was obtained when the treatment was carried out at 4 kV/cm at 10 Hz using 100 pulses of 10 μs. At these optimal conditions, predicted FaOH, FaDOH and FaDOAc contents relative to untreated carrot slices were 188%, 164.9% and 166.8%, respectively. The results of the optimisation of PEF parameters were validated by repeating the experiment at the conditions predicted. The predicted values were in close agreement with experimental values (Table 3) and were found to be not significantly different (*p* > 0.05). In addition, variations between the predicted and experimental values obtained for relative FaOH, FaDOH and FaDOAc -levels were within an acceptable error range as depicted by average mean deviation (*E*%, Table 3); therefore, the predictive performance of the established model may be considered acceptable.

**Table 3:** Average mean deviation (*E*%) values of predicted and actual values at optimal mild PEF treatment conditions set up at 4 kV/cm with 100 number of pulses of 10-μs and at 10 Hz of pulse frequency.

	Actual Values at Optimal PEF	Predicted Values at Optimal PEF	*E* (%)	Desirability
**FaOH**	195 ± 6	188.0	3.58	0.910
**FaDOH**	170 ± 5	164.9	2.98	0.910
**FaDOAc**	168 ± 2	166.8	0.68	0.910

## 3. Experimental Section

### 3.1. Chemicals

Acetonitrile, ethyl acetate and HPLC grade water (99%) were obtained from Sigma-Aldrich (Arklow, Ireland). Diatomaceous earth and silica gel (SiO_2_) were purchased from ThermoFisher Scientific (Dublin, Ireland).

### 3.2. Preparation of Carrot Slices

Whole carrots (*Daucus carota*, cv. Nerac) were purchased in a local market (Tesco, Dublin, Ireland). The carrots were washed with tap water and cut into slices of 1 cm thickness, retaining the peel. Only slices of around 3.6 ± 0.2 cm diameter were used for processing under PEF conditions; slices with smaller diameter were discarded. Carrot slices selected for the experiment consisted of a central stele (mostly vascular tissue) and a peripheral cortex layer.

### 3.3. PEF Conditions

PEF equipment used in this investigation was an ELCRACK^®^ HVP 5 unit (DIL, Quakenbrück, Germany) working in batch mode. A parallel-plate treatment chamber consisting of two stainless steel electrodes (total electrode area of 16 cm^2^ and gap of 1 cm) was used. The carrot electrode contact was around 5.65 ± 0.02 cm^2^. The apparatus generated square waveform pulses in bipolar mode. Carrot slices were treated at electric fields from 1 to 4 kV/cm using a number of pulses ranging from 100 to 1500 pulses, pulse frequencies from 10 to 200 Hz and pulse widths between 10 to 30 μs at ambient (~15–23 °C) temperature. Five (n = 5) carrot slices of around 69 g each were exposed to each PEF treatment and three replicates were processed per treatment.

Temperature of carrot slices after each PEF treatment was recorded with fibre optic temperature probes (Lumasense technologies Fluoroptic^®^ Temperature Probe, Santa Clara, CA, USA). Measurement of temperature was taken directly on the sample before and after each treatment, which did not exceed beyond 33.5 °C.

The specific energy depends on the voltage applied, treatment time, and resistance of the treatment chamber that varies according to the geometry and conductivity of the material treated [13]. The specific energy per pulse (*W*') was calculated by following Equation (4):

(4) $$W\prime =\frac{1}{\rho }\int_{0}^{\infty }K\cdot E_{\left(t\right)}^{2}d\tau$$

where ρ (kg/m^3^) is the density of the treated product; K is the electrical conductivity (S/m), E (V/m) is the electric field strength; and *τ* (s) is the pulse width. The total specific energy applied (*W*) was calculated by multiplying the energy per pulse (*W*') by the number of pulses.

PEF-treated slices were then frozen at −80 °C followed by lyophilisation for 18 h in a freeze-drier (D80 Leanne Freeze Drier, Cuddon, New Zealand) with a temperature of 30 °C and a pressure of 0.02 mbar.

### 3.4. Extraction of Polyacetylenes

Extraction of polyacetylenes was performed using pressurized liquid extraction with an ASE 200 automated system (Dionex, Surrey, UK) as described previously [11]. The optimized conditions were 100% ethyl acetate at a pressure of 800 psi, temperature off, for three static cycles. Freeze-dried carrot slices were milled to powder using a Retsch Mixer Mill (Retsch MM400, Haan, Germany) and 1 g was mixed with 4 g of SiO_2_ Hyflow diatomaceous Earth placed in a 22 mL ASE extraction cell. The remaining volume of the cell was filled with diatomaceous earth. The extracts were dried under N_2_ at room temperature using a Techne Sample Concentrator (Techne DRIBLOCK DB-3D, Staffordshire, UK) and the residues re-diluted in 1.5 mL of acetonitrile. The re-diluted residues were centrifuged (13,000 rpm for 1 min) and transferred to a 2 mL amber vial prior to RP-HPLC analysis.

### 3.5. Separation and Quantification of Polyacetylenes

Separation and quantification of polyacetylenes was carried out by RP-HPLC analysis using an Agilent 1100 series HPLC system (Agilent Technologies, Boeblingen/Stuttgart, Germany) equipped with multiwavelength UV detector. Chromatographic conditions used were as described by Søltoft *et al.* [23] with some modifications. The chromatographic separation was carried out on a Luna 5 μ C18, 4.6 × 100 mm column (Phenomenex, Torrance, CA, USA) at a flow rate of 1.0 mL/min at 40 °C, with an injection volume of 10 μL. The A and B eluents were Milli-Q water and acetonitrile, respectively. The gradient program was as follows: 70% B for 5 min, a linear gradient to 86% B for 18 min, a linear gradient to 100% B for 2 min, isocratic elution for 10 min, followed by a 3 min ramp back to 70% B and re-equilibration for 4 min, giving a total run time of 38 min. Runs were monitored using a UV-visible detector at 205 nm. Polyacetylenes were identified by addition of standard and quantified using external standard calibration curves. The standards (10–60 μg/mL) used in all cases were isolated from a large scale ethyl acetate extraction of freeze-dried carrots and purified, in house, using optimized column chromatography and preparative HPLC methods described by Rawson *et al.* [9]. Results were expressed as µg of each individual polyacetylene per g of dry weight (DW) of carrot. Relative polyacetylene content was used to describe the changes in the content of PEF-treated carrot slices (Equation (5)):

(5) $$\text{Relative polyacetylene content}(\%)=\frac{C_{t}}{C_{0}}x100$$

where *C*_t_ and *C*_0_ are the concentration of polyacetylenes of treated and untreated samples, respectively.

### 3.6. Experiment Design

A face-centred central composite response surface analysis was used to determine the effect of electric field strength (*E*), number of pulses (n), pulse frequency (ƒ) and pulse width (*τ*) on the polyacetylene content of carrot. Independent variables were chosen at the following levels: *E* (from 1 to 4 kV/cm), n (from 100 to 1500), *f* (from 10 to 200 Hz) and *τ* (from 10 to 30 μs). The experimental design was performed in triplicate (Table 1). A polynomial response surface was fitted to experimental data. The second-order response function was predicted by Equation (6):

(6) $$Y=\beta_{o}+\sum_{i=1}^{3}\beta_{i}X_{i}+\sum_{i=1}^{3}\beta_{ii}X_{i}^{2}+\sum_{i=1}^{2}\sum_{j=i+1}^{3}\beta_{ij}X_{i}X_{j}$$

where *Y* is the dependent variable, *β_o_*, *β_i_*, *β_ii_* and *β_ij_* are the constant, linear, quadratic and interaction regression coefficients, respectively and *X_i_* represent the encoded values of the variables. The non significant terms were deleted from the second-order polynomial model after an ANOVA test, and a new ANOVA was performed to obtain the coefficients of the final equation for better accuracy. Design Expert 7.0 software (Stat Ease Inc., Minneapolis, MN, USA) was used to generate quadratic models that fit the experimental data and to draw the response surface plots.

### 3.7. Model Validation

The predictive performance of the developed models describing the combined effect of electric field strength (X_1_), number of pulses (X_2_), pulse frequency (X_3_) and pulse width (X_4_) on independent variables (FaOH, FaDOH and FaDOAc) of carrot slices were valided with optimal PEF treatment conditions as predicted by the design.

The criterion used to characterize the fitting efficiency of the data to the model was the multiple correlation coefficients (R^2^) and the average mean deviation (Equation (7)):

(7) $$E(\%)=\frac{1}{n_{e}}\sum_{i=1}^{n}\|\frac{V_{E}-V_{P}}{V_{E}}\|\times 100$$

where *n*_e_ is the number of experimental data, *V*_E_ is the experimental value and *V*_P_ is the predicted value.

## 4. Conclusions

The PEF pre-treatment of carrot slices was effective to enhance pressurized-liquid extraction of polyacetylenes. Optimal PEF process conditions with regard to electric field strength (1–4 kV/cm), number of pulses (100–1500), pulse frequency (10–200 Hz) and pulse width (10–30 μs) were identified using response surface methodology (RSM). The results demonstrated that the combined treatment of 4 kV/cm, 100 number of pulses of 10 μs at 10 Hz was optimal for maximising FaOH, FaDOH and FaDOAc levels. Data fitted significantly (*p* < 0.0001) the proposed second-order response functions. Therefore, PEF is a promising technique to increase extraction of polyacetylenes from minimally process fresh cut carrots such as carrot slices.
